# Commentary: Meta-analysis overstates benefit of antidepressant combination therapy with α2-antagonists and reuptake inhibitors in major depression

**DOI:** 10.3389/fpsyt.2023.1164471

**Published:** 2023-05-11

**Authors:** Jonathan Henssler, Tom Bschor, Christopher Baethge

**Affiliations:** ^1^Department of Psychiatry and Psychotherapy, St. Hedwig-Krankenhaus, Charité–Universitätsmedizin Berlin, Freie Universität Berlin and Humboldt-Universität zu Berlin, Berlin, Germany; ^2^Department of Psychiatry and Psychotherapy, University of Cologne Medical School, Cologne, Germany; ^3^Department of Psychiatry and Psychotherapy, University Hospital of Dresden, Dresden, Germany

**Keywords:** antidepressants, response letter, antidepressant combination, meta-analysis, depression

We appreciate Dr. Kennedy's thoughts (1) on our meta-analysis of antidepressant combination RCTs (2) as they provide an opportunity to address what may be common concerns.

Based on sample size, Kennedy retrospectively singles out five studies he considers “robust”—without defining the term or the threshold—and he emphasizes smaller effects in those studies relative to the summary standardized mean difference (SMD) of 0.37 [0.19–0.55] across all 18 RCTs. While Kennedy repeatedly calls the five trials he picked negative, this is simply not true for their summary effect (SMD: 0.1 [0.04–0.17]) (*post-hoc* analysis, conducted for this response letter). However, it is not size that matters most in meta-analysis: Risk of bias is the key feature of trials. Importantly, therefore, in a prospectively planned analysis restricted to low risk of bias studies the effect size turned out to be practically identical: 0.36 [0.19–0.53; 11 studies].

In principle, following large RCTs rather than a meta-analysis for clinical decision making, as Kennedy proposes, is a debatable approach. However, it is a deviation from a cornerstone of evidence-based medicine and runs a high risk of bias, particularly when done retrospectively. In the present case, for example, four out of five studies that Kennedy prefers are limited or largely limited to patients with treatment resistant or chronic depression and the fifth trial excluded patients exhibiting early response. Clinically, efficacy of *any* treatment is expected to be smaller in this population of patients. In fact, it is one result of our original analysis (2) that this common finding applies to antidepressant combinations too (Table 2 in the original paper).

Kennedy reiterates our discussion of the sizable heterogeneity found in the main meta-analysis. Unfortunately, he fails to mention that heterogeneity drops below 50% in various secondary outcome analyses, e.g., in regard to treatment response (OR 1.49 [1.18–1.87], I-squared 48%), supporting the findings of our primary analysis. As an aside, tau-squared, a measurement of heterogeneity independent of meta-analysis sample size, indicates acceptable variability in trial results.

In our paper, we also discussed the potential of small study effects (as a result of possible publication bias) —another topic of concern for Kennedy—and we provided calculations of its possible impact on our results. Nevertheless, in RCTs comparing antidepressant combination to monotherapy, publication bias, while a statistical possibility, appears much less likely than in placebo-controlled trials of antidepressant drugs. Indeed, it cannot be discounted that, regardless of statistical significance, 17 out of 18 RCTs resulted in stronger antidepressant effects of combination versus monotherapy – including all five trials selected by Kennedy, virtually excluding a role of chance (*p* = 0.0001, sign test). As a matter of fact, even the one diverging study cannot be considered negative with regard to combinations because responder rates were generally large and combination treatment was more acceptable to patients than high-dose imipramine monotherapy (3).

Finally, in an earlier paper we have provided evidence why the specific superiority of a reuptake inhibitor and α2-antagonist combinations results from synergistic interaction and is not due to a simple dose effect (4). Still, the effect size we arrived at is an estimate and, obviously, may be changed by future studies. Also, the confidence interval indicates a relatively wide range of possible average effects, but in discussing the significance of our estimate for clinical practice it must not be forgotten that, in the trials included, combination treatment has not been evaluated against placebo but against antidepressant monotherapy, that is, against a treatment of shown efficacy. In summary, even small incremental effects may prove to be valuable for patients.

## Author contributions

JH and CB: conceptualization, data curation, formal analysis, investigation, interpretation, methodology, project administration, resources, validation, visualization, writing original draft, and writing review and editing. TB: conceptualization, investigation, interpretation, methodology, resources, supervision, validation, and writing review and editing. All authors approved the final version of the manuscript.
